# Van Wyk-Grumbach syndrome and oligosyndactyly in a 6-year-old girl: a case report

**DOI:** 10.1186/s13256-020-02472-z

**Published:** 2020-09-16

**Authors:** Niranjalee Samanthika Egodawaththe, Sumudu Nimali Seneviratne, Suvini Gunasekara, Sathika Manori Amarasekara, Kumudu Weerasekara

**Affiliations:** 1 Lady Ridgeway Hospital for Children, Colombo, Sri Lanka; 2 Department of Paediatrics, Faculty of Medicine, University of Colombo, Colombo, Sri Lanka

**Keywords:** Van Wyk-Grumbach syndrome, Precocious puberty, Hypothyroidism, Oligosyndactyly

## Abstract

**Background:**

Van Wyk-Grumbach syndrome refers to the development of isosexual precocious pseudopuberty and multicystic enlarged ovaries in the presence of hypothyroidism and delayed bone age. It is a rare presentation of untreated hypothyroidism. The prepubertal response in Van Wyk-Grumbach syndrome is always isosexual and mediated by very high thyroid-stimulating hormone levels acting through the follicle-stimulating hormone receptors inducing a follicle-stimulating hormonal effect. Early recognition and thyroid hormone replacement can completely regress precocious puberty and ovarian enlargement, while improving the final height achievement.

Oligosyndactly is a congenital bony abnormality and can manifest either as an isolated malformation or as a component of a syndromic diagnosis. However, development of hypothyroidism in children with this peculiar bony deformity has rarely been described in the medical literature, with the exception of Cenani-Lenz Syndactyly syndrome.

**Case presentation:**

We report the case of a 6-year-old Sri Lankan girl who presented with a 2-day history of vaginal bleeding and exertional dyspnea. She had marked short stature (well below −3 standard deviations) with an upper segment to lower segment ratio of 1.47. This girl had isolated breast development of Tanner stage 2. She was diagnosed to have acquired hypothyroidism secondary to autoimmune thyroiditis and also had macrocytic anemia, pericardial effusion, gonadotropin-releasing hormone-independent precocious puberty with radiological evidence of pubertal changes in the uterus, and multicystic ovaries. Interestingly, she also had post-axial oligosyndactyly in both feet and right-sided clubfoot. The diagnosis of Van Wyk-Grumbach syndrome was made based on the clinical and laboratory features. Her symptoms were successfully managed with L-thyroxine therapy.

**Conclusions:**

Acquired hypothyroidism is a relatively common endocrine disorder among children and early recognition is important to prevent serious complications like Van Wyk-Grumbach syndrome. Sexual precocity with delayed bone age and stunting should direct our minds toward this unique diagnosis. It is always necessary to identify the other associated anomalies in addition to the primary diagnosis since these features may direct to a syndromic diagnosis.

## Background

Precocious puberty may be a rare presentation of untreated hypothyroidism, while delayed puberty is its norm [1]. Van Wyk-Grumbach syndrome (VWGS) refers to the development of isosexual precocious pseudopuberty and multicystic enlarged ovaries in the presence of hypothyroidism and delayed bone age [2]. The hormonal spectrum of these patients is characterized by elevated prolactin, estradiol, thyroid-stimulating hormone (TSH), together with decreased free thyroxine [3]. The prepubertal response in VWGS is always isosexual and mediated by very high TSH levels acting through the follicle-stimulating hormone (FSH) receptor inducing a follicle-stimulating hormonal effect [4]. Phenotypically, breast enlargement, multicystic ovaries, and menstrual bleeding are encountered in girls whereas boys only have testicular enlargement [3]. Early recognition and commencement of thyroid hormone replacement are the key steps in the management of patients with VWGS. These measures will resolve symptoms rapidly and improve the height achieved. In addition, reversal to the prepubertal state is typically seen following the appropriate treatment. Oligosyndactly is a congenital bony abnormality and can manifest either as an isolated malformation or as a component of a syndromic diagnosis. However, development of hypothyroidism in syndromic children with this peculiar bony deformity has rarely been described in the medical literature with the exception of the rare genetic disorder Cenani-Lenz Syndactyly (CLS) syndrome.

In this article, we report the clinical details of the case of a Sri Lankan girl who had typical features of VWGS and exhibited a successful recovery. The aim of this report is to highlight the devastating consequences of longstanding hypothyroidism and the importance of timely intervention to preserve the final height of the affected children as there can be diagnostic dilemma for this disease at the outset. Moreover, we review the literature to clarify whether there is an unusual association between VWGS and oligosyndactyly, which has not been described previously.

## Case presentation

A 6-year-old girl from central Sri Lanka presented with a 2-day history of vaginal bleeding. There was no history of local trauma and other bleeding manifestations. She had recent onset exertional dyspnea. Her parents reported that the child was not performing well academically and was not interacting with her peers. There was no galactorrhea, headache, or visual disturbance. She was the second child born to healthy unrelated parents, with an uneventful birth and perinatal history. There was no family history of pubertal precocity or thyroid disease. A detailed dietary history included a 24-hour dietary recall and revealed that her diet was highly deficient in both quality and quantity and she had only received about 60% of the required calories. Our patient had been lost to follow-up at the routine field pediatric clinic when she was 3 years.

**Fig. 1:**
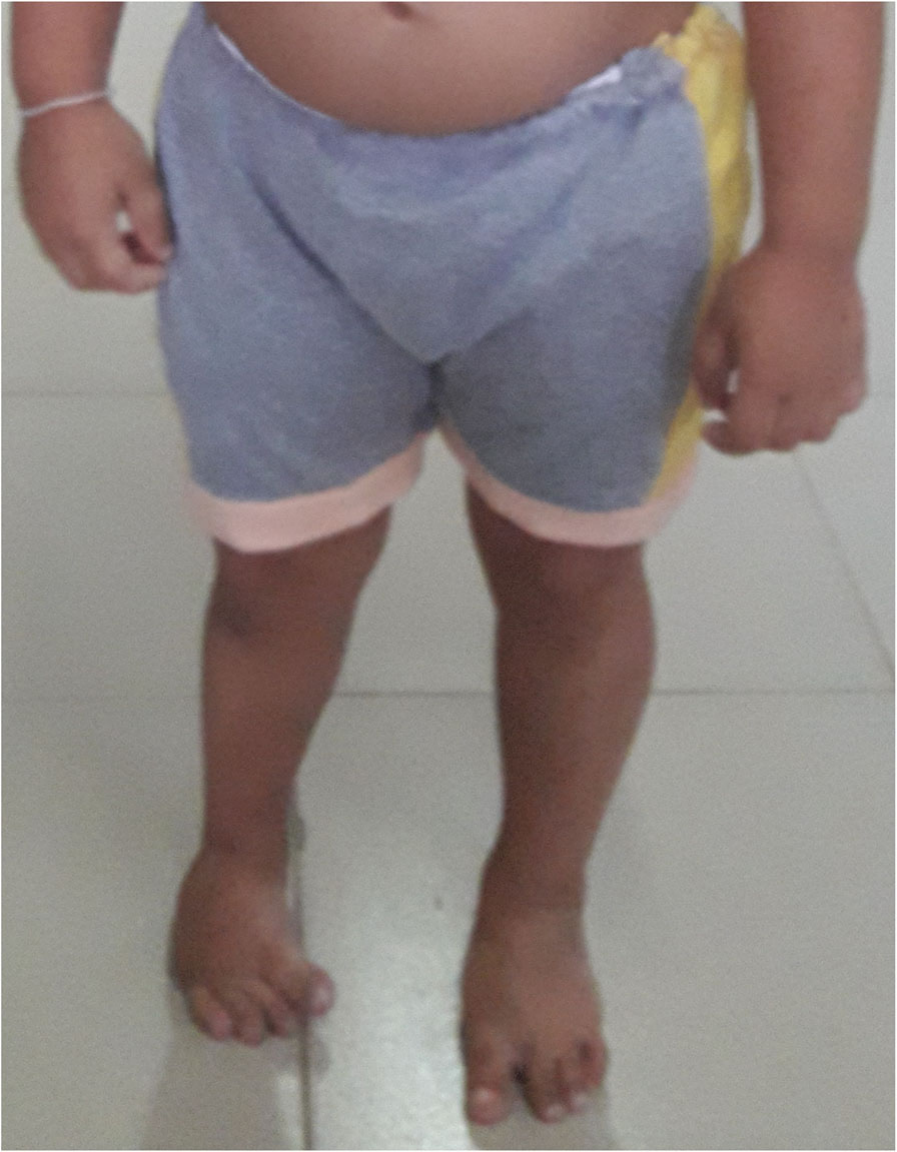
Phenotypic appearance showing right-sided talipes deformity with bilateral post-axial oligosyndactyly

On examination, the child had disproportionate short stature for her age (height 84 cm, well below −3 standard deviation score (SDS)) with an upper segment to lower segment ratio 1.47 (normal 1.1). Her weight was 13 kg (below the third centile) and her head circumference was 45 cm (<−3SDS). She had post-axial oligosyndactyly in both feet and right-sided talipes deformity (Fig. 1).

She was lethargic and pale. There was no goiter. She had bradycardia with a pulse rate of 64 beats/minute. Her blood pressure was normal while her heart sounds were muffled. Her respiratory rate at rest was 16 cycles/minute and oxygen saturation on air was 100%. She had early breast development (Tanner stage 2), with no axillary or pubic hair. Abdominal, respiratory, and nervous system examinations were normal, except for slow-relaxing reflexes.

**Fig. 2:**
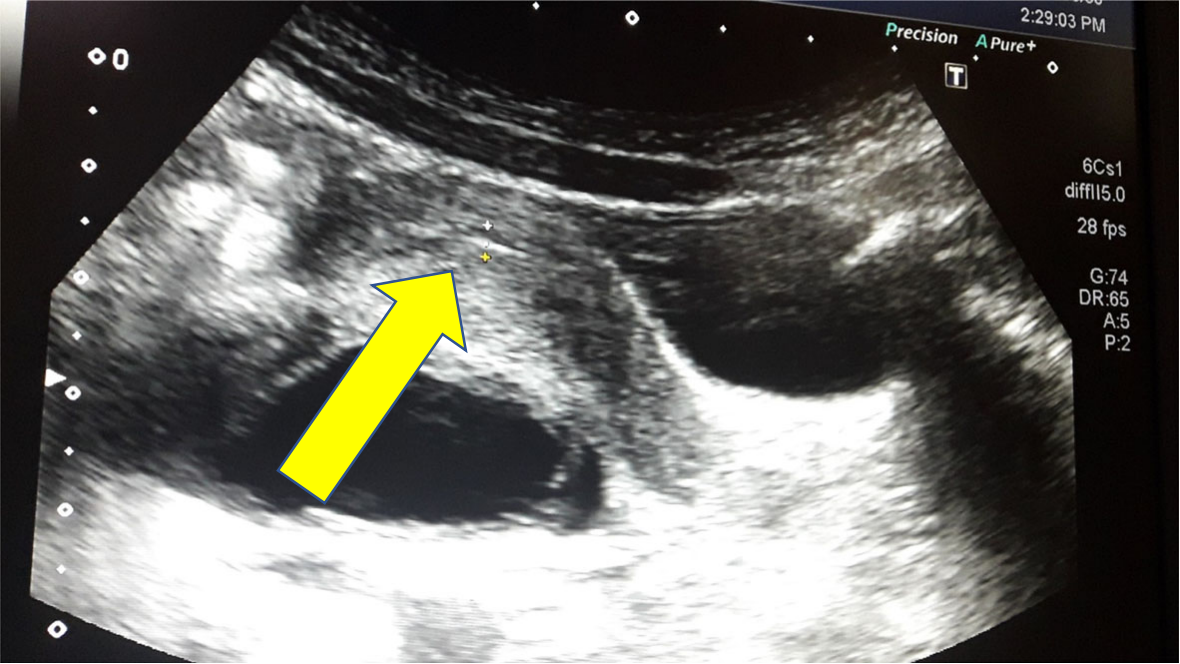
Ultrasound scan image of enlarged uterus (yellow arrow) measuring 3.6 cm × 1.9 cm × 2.5 cm and endometrial thickness of 4 mm

**Fig. 3:**
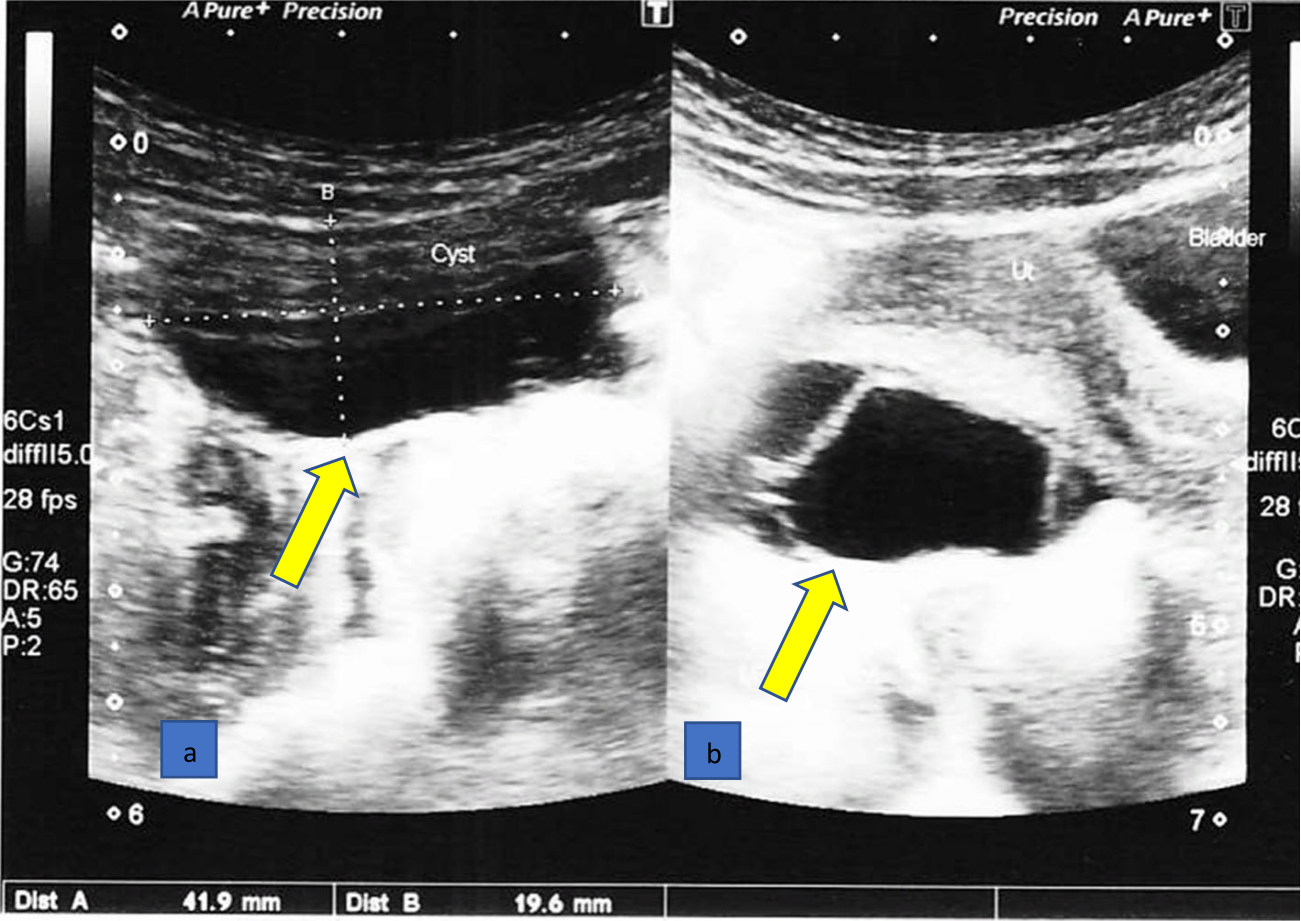
Ultrasound scan image of a 3.3 cm × 2.1 cm × 2.5 cm sized complex cyst (yellow arrow) (**a**) and enlarged left ovary (yellow arrow) (**b**)

On investigation, her bone age was delayed (3 years), and laboratory test results (Table 1) were suggestive of autoimmune hypothyroidism, macrocytic anemia, and gonadotropin-releasing hormone (GnRH)-independent precocious puberty. An ultrasound scan of her pelvis showed an enlarged uterus with pubertal changes (size 3.6 cm × 1.9 cm × 2.5 cm, fundus: cervix ratio 3.6:2.7, endometrial thickness (ET) 4 mm) (Fig. 2), and enlarged ovaries with multiple cysts (Fig. 3). She had an ultrasonically normal renal system. Thyroid ultrasonography had features consistent with thyroiditis, namely, a mildly enlarged gland with irregular echo patterns and high vacularization. Two-dimensional echocardiography revealed a moderate pericardial effusion, which had caused the recent-onset exertional dyspnea. An abnormally wide and deep sella turcica was seen on a lateral skull X-ray. Magnetic resonance imaging of her brain to evaluate the pituitary gland in detail was not done due to limited radiological facilities. There were no radiological bony abnormalities in her lower limbs apart from oligosyndactyly of the second and third toes bilaterally. A formal developmental assessment was performed by a child psychiatrist and found that her intelligence quotient (IQ) was normal. Audiological and visual assessment results were normal.

**Table 1:** Blood test results on admission

Test	Initial results	Reference range
Free thyroxine	< 5.15	9–25 pmol/L
TSH	> 100	0.7–4.61 mIU/L
TPO Ab titer	495.64	< 5.61 IU/L
TG Ab titer	231.63	< 4.11 IU/L
LH	< 0.07	0.08–3.9 IU/L
FSH	8.72	0.1–11.3 IU/L
Estradiol	416	< 59 pmol/L
Prolactin	966	109–556 mIU/L
Hemoglobin	9	11.5–14.5 g/dL
Cortisol	247	83–580 nmol/L
Mean corpuscular volume	98	76–90 fL
ESR	10	< 20

*ESR* erythrocyte sedimentation rate, *LH* luteinizing hormone, *TG Ab* thyroglobulin antibody, *TPO Ab* thyroid peroxidase antibody

On the basis of the clinical and laboratory findings, she was diagnosed to have VWGS. She was initially commenced on a low dose of L-thyroxine at 5 μg/kg/day to avoid precipitating cardiac failure and the dose was gradually titrated up, in accordance with the clinical and biochemical parameters. The age-appropriate replacement dose was thereby achieved gradually. Before starting treatment, her serum cortisol level (8 am value) was analyzed and confirmed to be normal.

**Fig. 4:**
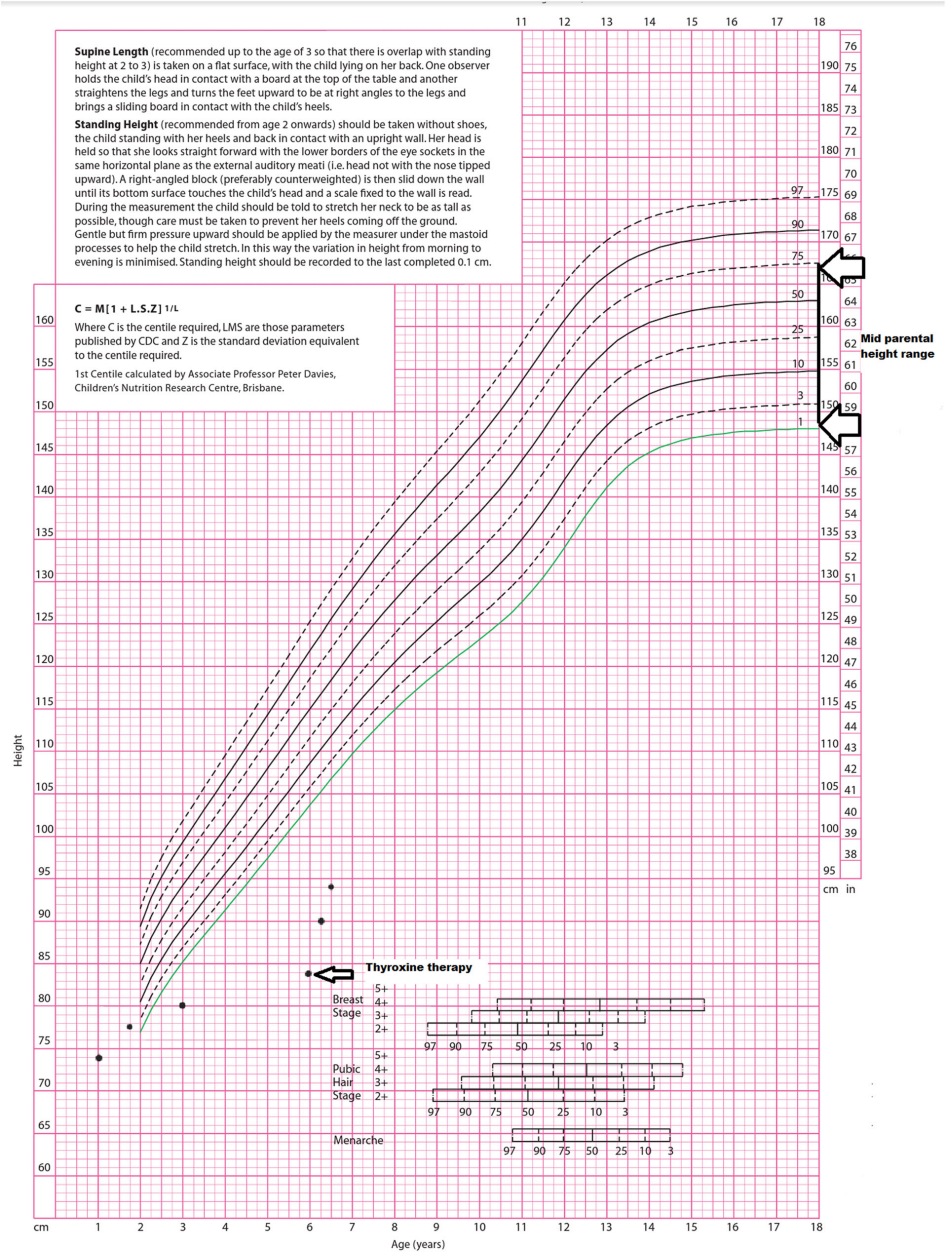
Growth chart of our patient showing height well below 3 standard deviations at presentation and rapid acceleration following thyroid hormone replacement

On follow-up, she exhibited marked improvement in clinical parameters. Her height increased by 6 cm over the first 3 months and another 5 cm over the subsequent 3 months, consistent with a height velocity surpassing the 97th percentile (Fig. 4). The echocardiogram done following the first month of thyroxine therapy revealed complete resolution of her pericardial effusion and she had recovered from the exertional dyspnea and bradycardia by 2 weeks after the treatment start. Her anemia was resolved following 1 month of thyroxine. Furthermore, her ovarian cysts disappeared and breast size regressed, and she did not experience further menstruation. She started to perform school activities enthusiastically and her social interactions were improved.

## Discussion and conclusions

In 1960, Van Wyk and Grumbach first described this unusual clinical spectrum and it is now termed VWGS [2]. The incongruous combination of precocious puberty and delayed growth is characteristic of VWGS. The presence of short stature and delayed bone age and the lack of axillary and pubic hair development differentiates this condition from other causes of pubertal precocity [1, 5].

The etiology of hypothyroidism in VWGS is often lymphocytic thyroiditis, though it also has been reported in association with unrecognized congenital hypothyroidism [6, 7]. In this child, elevated thyroid autoantibodies and preserved IQ pointed to acquired autoimmune hypothyroidism, though it is relatively uncommon at such a young age [8].

The hormonal interaction involving the pathogenesis of VWGS is complex and speculated. The most acceptable theory is that the very high TSH level seen in profound hypothyroidism interacts with the FSH receptor. Gonadotropins as well as TSH are glycoproteins and all these hormones share the same α-subunit, though the β-subunit is identical to each hormone. Anasti *et al.* have demonstrated the interaction of recombinant TSH with FSH receptor to stimulate adenylate cyclase activity and shown that recombinant TSH acted as a competitive inhibitor of FSH [3, 9]. Therefore, increased levels of TSH act through the FSH receptors (“specificity spillover”) and induce FSH-like effects on the gonads, causing multicystic ovaries, uterine enlargement with bleeding and breast enlargement in girls [5, 7, 9–11]. In boys, this leads to macroorchidism without virilization [10]. In addition, persistently high thyrotropin-releasing hormone (TRH) stimulates FSH secretion. Patients with VWGS have a high or upper limit of the age-appropriate level of FSH and we noticed a high FSH level in our patient. Axillary and pubic hair growth does not occur, as there is no stimulation of adrenarche, which explains the absence of consonant pubertal development in VWGS.

The role of prolactin theory elaborates the discordance between FSH and luteinizing hormone (LH) in this syndrome [4, 5]. Hyperprolactinemia occurs due to unopposed secretion of prolactin due to elevated TRH and prolactin can increase the sensitivity of the ovaries to gonadotropins, while slowing down gonadotropin-releasing hormone (GnRH) pulse frequency [5, 12]. Interestingly, slow GnRH pulses suppress LH while producing FSH [13]. This causes an isolated FSH response as in our patient.

In 2011, Durbin and colleagues exemplified VWGS in a 12-year-old Caucasian girl who also had a pituitary macroadenoma and bilateral ovarian masses mimicking a tumor [14]. Another typical phenotype of VWGS with coexisting TSH-secreting adenoma and hyperprolactinemia was described in an 8-year-old girl with autoimmune thyroiditis by Baranowski and Högler in 2012 [5]. Pituitary hyperplasia occurs secondary to thyrotroph hyperplasia, which is induced by lack of thyroid hormonal feedback [15]. In the setting of untreated prolonged hypothyroidism, it can progress to pituitary adenoma. Our patient however, being a classic example of VWGS, only had pituitary hyperplasia, which was analyzed only with a lateral skull X-ray and revealed a wide and deep sella turcica.

Hypothyroid children have slow growth and increased weight. Moderate to severe obesity is not typical but commonly observed. However, decreased growth velocity is a constant finding unlike the weight. Our patient was not complying with this rule as she was markedly underweight, probably contributed to by the diet and possible underlying syndromic diagnosis.

Anemia and pericardial effusion are also well described in untreated hypothyroidism. Hypothyroidism may lead to macrocytic anemia due to suppressed bone marrow activity and erythropoietin secretion [8]. Reduced hematopoiesis in response to decreased metabolic demand, dietary deficiency, menorrhagia, and as a part of autoimmune cluster are other proposed mechanisms in the pathogenesis of anemia in hypothyroid patients [16]. Undetected and progressive hypothyroidism is associated with pericardial effusion and the attributed mechanisms are increase in capillary permeability causing protein-rich fluid extravasation into the pericardial sac, impaired lymphatic drainage, and increased salt and water retention [17].

In addition to the features consistent with VWGS, our patient had oligosyndactyly, right sided clubfoot, microcephaly, and severe growth failure, directing us to a possible syndromic diagnosis. Webbing of adjacent digits with or without bony fusion is termed as syndactyly and it is a common hereditary limb anomaly. It can occur either as an isolated malformation or as a component of a syndromic diagnosis and more than 300 associated syndromes have been identified [18]. Oligosyndactyly is one of the syndactyly phenotypes and it is a frequent feature in several syndromes, namely, CLS syndrome, fibular aplasia, tibial campomelia and oligosyndactyly (FATCO) syndrome, fibular hemimelia (FH), Cornelia de Lange (CdL) syndrome and chromosome 2q35 duplication syndrome. A comprehensive literature review revealed the rare association of syndactyly with hypothyroidism in CLS syndrome [19, 20]. The genetic etiology of this is mapped to CLC1 locus on chromosome region 11p11.2-q13.1 and is thought to result from mutations in the LRP4 gene [20]. CLS syndrome is characterized by syndactyly and oligodactyly of fingers and toes, facial dysmorphism, renal anomalies and congenital hypothyroidism [20, 21]. However, the absence of dysmorphism and highly elevated thyroid autoantibody titers suggest the index case is a remote possibility of this diagnosis.

FATCO syndrome is a descriptive term to a collection of congenital limb deficiencies as described above. It has a great clinical variability and is sporadic in origin [22]. Patients with FATCO syndrome have normal IQ levels and not dimorphic or other malformations [22, 23]. FH is a spectrum of limb anomalies from mild fibular hypoplasia to fibular aplasia. It can occur sporadically or as a part of a syndrome and the anomalies are usually unilateral [22]. Associated components of FH are femur and tibia shortening, clubfoot, vulgus deformity, and anteroposterior instability of the knee and ankles [22, 24]. CdL syndrome is a dominantly inherited disorder and is characterized by facial dimorphism, limb anomalies, microcephaly, somatic growth, and cognitive retardation [25]. Chromosome 2q35 duplication syndrome also has autosomal dominant inheritance and the characteristic features are craniosynostosis and syndactyly [26]. However, occurrence of autoimmune thyroiditis has never been described in any of these syndromes.

Acquired hypothyroidism is a relatively common endocrine disorder among children, and early recognition is important to prevent serious complications like VWGS and pericardial effusions. Moreover, VWGS often comprises a diagnostic dilemma because of its rarity, and pubertal delay is the well-known consequence of prolonged untreated hypothyroidism. But its identification has paramount importance since the pubertal precocity is reversible and final height can be improved. Sexual precocity evident by isolated breast development in girls and testicular enlargement in boys occurring in a short-statured, obese child with delayed bone age would be the red flag signs of this rare diagnosis. In our patient’s case, the presence of oligosyndactyly is likely to be an isolated finding since she had no characteristic syndromic features. We hope that this case history will encourage clinicians to manage hypothyroid patients optimally and promptly to avoid its complications and will persuade them to identify associated unusual features, which may direct into a syndromic diagnosis.

## Data Availability

Not applicable.

## Abbreviations

CdL: Cornelia de Lange
CLS: Cenani-Lenz Syndactyly
ESR: Erythrocyte sedimentation rate
ET: Endometrial thickness
FATCO: Fibular aplasia, tibial campomelia and oligosyndactyly
FH: Fibular hemimelia
FSH: Follicle-stimulating hormone
GnRH: Gonadotropin-releasing hormone
IQ: Intelligence quotient
LH: Luteinizing hormone
TG Ab: Thyroglobulin antibody
TPO Ab: Thyroid peroxidase antibody
TRH: Thyroid-releasing hormone
TSH: Thyroid-stimulating hormone
SDS: Standard deviation score
VWGS: Van Wyk-Grumbach syndrome

## References

[1] Asirvatham AR, Mahadevan S, Balachandran K, Balasubramaniam SK. Van Wyk Grumbach syndrome: a case series and review of literature. Int J Sci Res. 2018;7(2):19–20.

[2] Van Wyk JJ, Grumbach MM. Syndrome of precocious menstruation and galactorrhoea in juvenile hypothyroidism: an example of hormonal overlap in pituitary feedback. J Pediatr. 1960;57:416–35.

[3] Zhang S, Yang J, Zheng R, Jiang L, Wei Y, Liu G. Van Wyk-Grumbach syndrome in a male pediatric patient: a rare case report and literature review. Exp Ther Med. 2017;13(3):1151–4. 10.3892/etm.2017.4086 PMC5403655 28450956

[4] Indumathi CK, Bantwal G, Patil M. Primary hypothyroidism with precious puberty and bilateral cystic ovaries. Indian J Pediatr. 2007;74:781–8. 10.1007/s12098-007-0140-9 17785906

[5] Baranowski E, Högler W. An unusual presentation of acquired hypothyroidism: the Van Wyk-Grumbach syndrome. Eur J Endocrinol. 2012;166:537–42. 10.1530/EJE-11-0494 22170796

[6] Asami T, Kikuchi T, Kamimura S, Kinoshita S, Uchiyama M. Precocious puberty in a girl with congenital hypothyroidism receiving continuous L-thyroxine-replacement therapy. Pediatr Int. 2001;43(1):87–90. 10.1046/j.1442-200x.2001.01318.x 11208009

[7] Razi SM, Gupta AK, Gupta DC, Gutch M, Gupta KK, Usman SI. Van Wyk-Grumbach syndrome with Kocher-Debré-Sémélaigne srndrome: case report of a rare association. Eur Thyroid J. 2017;6:47–51. 10.1159/000448993 PMC5465749 28611948

[8] Marzuilla P, Grandone A, Perrotta, Ruggiero L, Capristo C, Luongo C, *et al.* Very early onset of autoimmune thyroiditis in a toddler with severe hypothyroidism presentation: a case report. Ital J Pediatr. 2016;42:61. 10.1186/s13052-016-0270-7 PMC4912771 27316517

[9] Anasti JN, Flack MR, Froehlich J, Nelson LM, Nisula BC. A potential novel mechanism for precocious puberty in juvenile hypothyroidism. J Clin Endocrinol Metab. 1995;80:276–9. 10.1210/jcem.80.1.7829625 7829625

[10] Sharma D, Dayal D, Gupta A, Saxena A. Premature menarche associated with primary hypothyroidism in a 5.5 year old girl. Case Rep Endocrinol. 2011;2011:678305. 10.1155/2011/678305 PMC3420754 22937289

[11] Wormsbecker A, Clarson C. Acquired primary hypothyroidism: vaginal bleeding in a quiet child. Can Med Assoc J. 2010;182960:588–99. 10.1503/cmaj.090883 PMC2845688 20176756

[12] Cetinkaya S, Sagsak E, Erdeve S, Aycan Z, Keskin M. Premature menarche associated with Hashimoto thyroiditis at 2 years 9 months: case report. Thyroid Disorders Ther. 2014;3:159.

[13] Thackray VG, Mellon PL, Coss D. Hormones in synergy: regulation of the pituitary gonadotropin genes. Mol Cell Endocrinol. 2010;314:192–203. 10.1016/j.mce.2009.09.003 PMC2815122 19747958

[14] Durbin KL, Diaz-Montes T, Loveless MB. Van Wyk Grumbach Syndrome: an unusual case and review of literature. J Pediatr Adolesc Gynecol. 2011;24(4):e93–6. 10.1016/j.jpag.2010.08.003 21600802

[15] Passeri E, Tufano A, Locatelli M, Lania AG, Ambrosi B, Corbetta S. Large pituitary hyperplasia in severe primary hypothyroidism. J Clin Endocrinol Metab. 2011;96(1):22–3. 10.1210/jc.2010-2011 21209043

[16] Chu JY, Monteleone JA, Peden VH, Graviss ER, Vernava BS. Anaemia in children and adolescents with hypothyroidism. Clin Pediatr. 1981;20:696–9. 10.1177/000992288102001102 7297010

[17] Purkait R, Prasad A, Bhadra R, Basu A. Massive pericardial effusion as the only manifestation of primary hypothyroidism. J Cardiovasc Dis Res. 2013;4:248–50. 10.1016/j.jcdr.2014.01.001 PMC3953684 24653591

[18] Deng H, Tan T. Advances in the molecular genetics of non-syndromic syndactyly. Curr Genomics. 2015;16:183–93. 10.2174/1389202916666150317233103 PMC4460222 26069458

[19] Kariminejad A, Stollfuβ B, Li Y, Bögershausen N, Boss K, Hennekam RC, *et al.* Severe Cenani-Lenz syndrome caused by loss of LRP4 function. Am J Med Genet. 2013;161A(6):1475–9. 10.1002/ajmg.a.35920 23636941

[20] Hettiaracchchi D, Bonnard C, Jayawardana SMA, Jin Ng AY, Tohari S, Venkates B, *et al.* Cenani-Lenz Syndactyly syndrome - a case report of a family with isolated syndactyly. BMC Med Genet. 2018;19:125. 10.1186/s12881-018-0646-1 PMC6057103 30041615

[21] JJarbhoua H, Hamamya H, Al-Hadidyb A, Ajlounia K. Cenani–Lenz syndactyly with facial dysmorphism, hypothyroidism, and renal hypoplasia: a case report. Clin Dysmorphol. 2008;17:269-70. 10.1097/MCD.0b013e328306a6ed 18978656

[22] Smets G, Vankan Y, Demeyere A. A female newborn infant with FATCO syndrome variant (fibular hypoplasia, tibial campomelia, oligosyndactyly) – a case report. J Belgian Soc Radiol. 2016;100(1):41–4. 10.5334/jbr-btr.929 PMC6100663 30151452

[23] Wilcow W, Coulter CP, Schmitz ML. Congenital limb deficiency disorders. Clin Perinatol. 2015;42(2):281–300. 10.1016/j.clp.2015.02.004 26042905

[24] Stevens P, Arms D. Postaxial hypoplasia of the lower extremity. J Pediatr Orthop B. 2000;20(2):166–72. 10739276

[25] Hulinsky R, Byrne JBL, Lowichik A, Viskochil DH. Fetus with interstitial del (5)(p13.1p14.2) diagnosed postnatally with Cornelia de Lange syndrome. Am J Med Genet. 2005;137A:336–8. 10.1002/ajmg.a.30856 16086407

[26] Bosse K, Betz RC, Lee YA, Wienker TF, Reis A, Kleen H, *et al.* Localization of a gene for syndactyly type a to chromosome 2q34-q36. Am J Hum Genet. 2000;67:492–7. 10.1086/303028 PMC1287194 10877983

